# Occlusal Relief and Dental Wear Predict Pulp Chamber Volume in Captive Baboons

**DOI:** 10.1002/ajpa.70375

**Published:** 2026-10-30

**Authors:** Myra F. Laird, Diego A. Contreras, Leslea J. Hlusko, Kristin L. Krueger

**Affiliations:** ^1^ Department of Basic and Translational Sciences University of Pennsylvania Philadelphia Pennsylvania USA; ^2^ Department of Anthropology McGill University Montreal Quebec Canada; ^3^ National Research Center on Human Evolution (CENIEH) Burgos Spain; ^4^ Department of Anthropology Loyola University Chicago Chicago Illinois USA

**Keywords:** aging, dental topography, dentin, molars, primates

## Abstract

Primates have adaptations that mitigate the functional consequences of dental wear. One such adaptation is dentin deposition within the pulp chamber, which may extend tooth functionality. Because this deposition can reflect age‐related secondary dentin and stimulus‐driven tertiary dentin, it remains unclear whether the resulting pulp volume reduction primarily tracks cumulative wear, chronological age, or aspects of occlusal morphology. Here, we test covariation among pulp chamber volume, dental wear, and dental topography in captive baboons. MicroCT and occlusal scans were used to evaluate relationships among pulp volume, dental wear, and dental topography for *M*_1_–*M*_3_ from 46 adult baboons that were part of the Southwest National Primate Research Center. Ages of death were adjusted for molar eruption timing to calculate “wear age,” representing the duration each tooth was in occlusion. Wear age was positively correlated with dental wear metrics and showed weaker relationships with dental topography. Corrected pulp volume was negatively associated with wear age, dentin exposure ratio, and Krueger‐Scott scores, while relief index (RFI) was the only dental‐topography measure associated with pulp volume across molars. These results suggest that pulp chamber reduction in baboons reflects both cumulative wear and the functional effects of occlusal morphology. Because RFI in worn teeth reflects wear‐related changes in crown geometry, we hypothesize that variation in occlusal relief may also relate to differences in how mechanical loads are distributed across the crown. These proposed relationships remain to be tested directly. Patterns of pulp chamber remodeling may therefore vary across taxa with dental morphology and loading environment.

## 1 Introduction

Dental wear is a central focus in biological anthropology because teeth are abundant in both the archaeological and fossil records and preserve signals of age, diet, and life history. Patterns of dental wear are widely used for age estimation in forensic and bioarchaeological contexts (e.g., Gustafson 1950; Scott 1979; B. H. Smith 1984; Lovejoy 1985; Mays et al. 2022) and provide insight into feeding ecology and life history in extant primates (e.g., Cuozzo and Sauther 2006; Galbany et al. 2014; Stuhlträger et al. 2021). As dental wear accumulates throughout an individual's lifespan, it alters tooth morphology, occlusal function, and the internal structure of the tooth. At the same time, teeth possess a range of compensatory mechanisms that mitigate the functional consequences of wear, potentially obscuring signals related to age and diet. Here, we investigate covariation among pulp chamber volume, dental wear, and dental topography in a sample of captive baboons with known ages at death.

Although new enamel cannot form after eruption, primate teeth demonstrate several other adaptations that buffer the effects of wear. Behavioral and biomechanical adjustments can reduce loading on worn teeth. For example, humans with increased dental wear often exhibit reduced bite force and altered transverse jaw movements during mastication, reflecting sensorimotor feedback from the teeth and surrounding tissues (bite force‐Jain et al. 2013; but see Sterenborg et al. 2018; jaw movements‐Silvester et al. 2021). Additional positional changes can occur within the dental arcade. Teeth may shift positions along the tooth row through processes such as super‐eruption, mesial drift of the posterior teeth, and lingual tipping of the incisors (Kaifu et al. 2003). These changes within the oral cavity are thought to increase with age‐related wear to compensate for lost crown height and maintain occlusal contact.

Teeth also show internal responses to wear and aging. Reductions in pulp chamber volume result from two related but distinct processes: the gradual deposition of secondary dentin over the lifespan and the formation of tertiary dentin in response to localized pathologic injury or mechanical stress. Secondary dentin is deposited continuously after tooth eruption and is often associated with chronological age, although rates of deposition can vary with metabolic demands (Molnar et al. 1981). Secondary dentin deposition also varies across the tooth and may vary with functional loading and dental wear (Philippas 1961; Philippas and Applebaum 1966; Nudel et al. 2021). Histologically, exposed patent dentinal tubules decrease in diameter with age, accompanied by reduced dentin hypersensitivity, and are thought to relate to the deposition of both secondary and tertiary dentin (Carvalho and Lussi 2017). Tertiary dentin, in contrast, forms in response to localized stimuli to the pulp‐dentin complex, including pathological dentin exposure or concentrated occlusal loading (Philippas 1961; A. J. Smith 2000; Goldberg and Smith 2004; Sangwan et al. 2013; Selig et al. 2021, 2025). Both processes reduce pulp chamber volume but reflect different underlying biological mechanisms. In humans, pulp chamber reduction has been used as an indicator of chronological age (e.g., Drusini 1997; Gotmare et al. 2019; Ikeda et al. 1985; Kvaal et al. 1995; Salvatierra‐Gálvez et al. 2023), consistent with the role of secondary dentin deposition. In non‐human primates, however, recent work suggests that dental wear and diet may also influence pulp chamber volume, potentially through tertiary dentin deposition Selig et al. (2021, 2025). Pulp chamber volume may therefore reflect a combination of age‐related and mechanically induced processes.

Dental wear is also intricately linked with occlusal morphology. As enamel and dentin are removed through wear, the shape and relief of the occlusal surface change, which can affect chewing efficiency and tooth function. Quantitative measures of dental topography provide a way to capture these morphological changes even when traditional landmarks are lost through wear. Dental topographic analysis uses methods derived from Geographic Information Systems (GIS) to quantify functional aspects of tooth shape (Ungar and M'Kirera 2003; Ungar and Williamson 2000), and a series of metrics are used to characterize different aspects of occlusal surface geometry. Dirichlet normal energy (DNE) quantifies the curvature and sharpness of the occlusal surface and has been associated with dietary differences across a range of primates (e.g., Bunn et al. 2011; Ledogar et al. 2013; Pampush, Spradley, et al. 2016; Pampush et al. 2022; Winchester et al. 2014). However, the relationship between DNE and dental wear can vary depending on species and occlusal morphology (Berthaume et al. 2019; Winchester et al. 2014). For example, concave DNE values increase with wear in *Alouatta palliata* (Pampush, Spradley, et al. 2016), whereas no relationship was found in *Ateles* (Pampush et al. 2018). This difference partly reflects how DNE was calculated, and more recent work recommends the use of convex DNE (vDNE) to distinguish differences between blunt and sharp cusps (Pampush et al. 2022). The relief index (RFI), calculated as the ratio of crown surface area to its two‐dimensional occlusal footprint, captures variation in crown relief and generally decreases with dental wear (Boyer 2008; Glowacka et al. 2016; Morse et al. 2023). As a result of this calculation, RFI in worn teeth is not independent of dental wear, and instead reflects a combination of wear and functional signals. Orientation patch count rotated (OPCR) quantifies the number of patches with distinct orientations and has been linked to the number of potential shearing areas on the occlusal surface, with higher values associated with folivorous diets (Boyer et al. 2010, Evans et al. 2007; Godfrey et al. 2012, Ledogar et al. 2013). Comparative analyses across Papionini, including multiple species of *Papio*, show that these measures of dental topography capture distinct interspecific variability associated with dietary differences (Avià et al. 2022).

Despite growing interest in both dental topography and pulp chamber volume, relatively little work has examined how changes in occlusal morphology relate to internal tooth responses such as dentin deposition within the pulp chamber. Specifically, measures of dental topography such as relief may influence how forces are transmitted across the crown and may alter the magnitude or distribution of mechanical stimuli experienced by the pulp‐dentin complex. Most previous studies have examined these processes independently, focusing either on external patterns of wear or internal changes in pulp chamber morphology (e.g., Pampush, Spradley, et al. 2016; Pampush et al. 2018; Selig et al. 2021, 2025). Consequently, it remains unclear whether pulp chamber remodeling primarily reflects cumulative wear, functional loading, or broader aspects of tooth morphology. As pulp chamber reduction may reflect both secondary and tertiary dentin deposition, disentangling cumulative age‐related change from localized, mechanically induced responses is especially important.

Baboons are an ideal taxon for examining these relationships because their bilophodont molars possess relatively tall cusps and pronounced occlusal relief, producing substantial changes in occlusal morphology as wear progresses. In addition, the availability of known‐age individuals from captive colonies consuming relatively controlled diets allows the duration of occlusal function to be estimated directly. Together, these factors make it possible to evaluate how dental wear, dental topography, and pulp chamber volume covary within the same individuals.

Here, we examine wear‐related changes in dental topography and pulp chamber volume in a sample of baboon molars with known ages at death. We analyze *M*_1_, *M*_2_, and *M*_3_ to evaluate potential differences across the molar row. Because the *M*_1_ erupts earlier and experiences higher occlusal loads, it is expected to accumulate greater wear than the more distal molars (Edmonds and Glowacka 2020). Baboons are highly sexually dimorphic, but similar relationships between wear and tooth morphology are expected in males and females. We ask two questions:

*How do measures of dental topography and dental wear vary with wear age in baboons?*

We quantify multiple measures of dental topography and dental wear and define wear age as the amount of time the tooth was in occlusion. Increasing dental wear is expected to result in smaller values of vDNE, OPCR, and RFI and larger summed Krueger‐Scott scores (Krueger et al. 2025; Scott 1979) and dentin exposure ratio (DER) scores. Similar relationships are expected across the molar row, with larger changes occurring on the more mesial molars.

*Is pulp chamber volume more strongly associated with cumulative dental wear or with changes in dental topography?*

Because reductions in pulp chamber volume may reflect both secondary dentin accumulation and tertiary dentin deposition, size‐corrected pulp volume (CPV) is predicted to decline with wear age and dental wear. If occlusal morphology influences functional loading, however, measures of dental topography may explain additional variation beyond cumulative wear alone.

## 2 Materials and Methods

### 2.1 Sample

A sample of 46 (32 female, 14 male) captive, adult baboon skulls was obtained from the Southwest National Primate Research Center (SNPRC) in San Antonio, Texas (Table 1). This colony was founded in the 1970s, and the sampled animals lived between 1977 and 2006. The founding colony members were from several subspecies of *Papio hamadryas* (Hlusko et al. 2004; Cox et al. 2013). Subsequent revisions to baboon taxonomy elevated these subspecies differences to the level of species based on a phylogenetic species concept (Rowe and Myers 2016), although these baboon taxa still hybridize naturally at contact zones in the wild (Zinner et al. 2011). Accordingly, the individuals in this study are, to varying degrees, hybrids of *Papio anubis* , *P. cynocephalus* , and *P. hamadryas* (e.g., Hlusko et al. 2016; Thiebaut et al. 2026). All animals were born and raised in captivity, spending most of their time in large, outdoor group enclosures. Their diet primarily consisted of monkey chow with supplemental enrichment foods. The causes of death are unknown, but are unrelated to this study. All animals were necropsied and curated by Dr. James Cheverud, and the collection is now housed at the University of Illinois, Urbana‐Champaign. The individuals included in these analyses had well‐preserved dentition on at least one side that lacked chips, cracks, or other notable craniofacial pathological conditions.

**TABLE 1:** SNPRC number, age at death, wear age, and sex of the baboon sample.

SNPRC animal number	Sex	Age at death	M1 wear age	M2 wear age	M3 wear age
23	Female	16.07	14.41	12.32	9.91
39	Male	16.52	14.85	12.69	10.27
48	Male	14.96	13.30	11.13	8.71
49	Female	8.57	6.90	4.82	2.40
87	Male	15.66	13.99	11.83	9.41
163	Male	13.7	12.03	9.87	7.45
172	Female	13.19	11.53	9.44	7.03
174	Male	12.05	10.39	8.22	5.80
178	Female	12.73	11.06	8.98	6.56
189	Female	12.33	10.67	8.58	6.17
190	Female	10.96	9.29	7.21	4.79
196	Male	8.97	7.31	5.14	2.72
201	Male	11.81	10.15	7.98	5.56
224	Male	15.32	13.65	11.48	9.07
231	Female	14.62	12.95	10.87	8.45
253	Female	16.54	14.88	12.79	10.38
259	Female	14.01	12.34	10.26	7.84
260	Female	12.58	10.92	8.83	6.42
265	Female	15.16	13.50	11.41	8.99
281	Female	15.06	13.40	11.31	8.90
288	Female	14.65	12.99	10.90	8.49
311	Female	15.13	13.46	11.38	8.96
312	Female	16.38	14.71	12.63	10.21
316	Male	24.46	—	20.63	18.21
317	Female	23.16	21.50	19.41	17.00
322	Female	24.47	22.81	20.72	18.31
330	Female	13.05	11.38	9.30	6.88
332	Female	13.85	12.18	10.10	7.68
361	Female	26.09	24.43	22.34	19.93
378	Female	23.74	22.07	19.99	17.57
382	Female	14.37	12.70	10.62	8.20
384	Female	22.34	20.67	18.59	16.17
387	Female	24.67	23.01	20.92	18.51
393	Female	24.16	22.50	20.41	18.00
394	Female	14.67	13.00	10.92	8.50
398	Female	18.44	16.77	14.69	12.27
400	Male	13.38	11.71	9.55	7.13
401	Female	14.41	12.74	10.66	8.24
405	Male	17.59	15.92	13.76	11.34
406	Male	20.55	18.89	16.72	14.30
412	Female	25.78	24.11	22.03	19.61
421	Male	22.02	20.36	18.19	—
424	Female	21.38	19.71	17.63	15.21
432	Male	15.28	13.62	11.45	9.03
443	Female	15.04	13.37	11.29	8.87
456	Female	13.09	11.42	9.34	6.92

Building on earlier studies of SNPRC baboon dental variation recorded from plaster dental casts (Hlusko and Mahaney 2009), each baboon was scanned with a MEDIT i700 to generate a high‐resolution 3D model of the dentition (Figure 1). These surface scans were used to calculate a dentin exposure ratio (DER), across the entire occlusal surface. DER was defined as the ratio of exposed dentin area to total occlusal surface area. This measure of DER differs from those that standardize exposed dentin areas to the entire crown in occlusal view (e.g., Pampush et al. 2024), and was calculated in the MEDIT Link Software (v 3.4.2, Medit, Seoul, Republic of Korea) using three‐dimensional meshes to measure surface area as described by Krueger et al. (2025). Wear scores were calculated from the intraoral scans using the Krueger‐Scott method, which assesses enamel loss across quadrants rather than at individual cusps (Krueger et al. 2025; Scott 1979).

**FIGURE 1:**
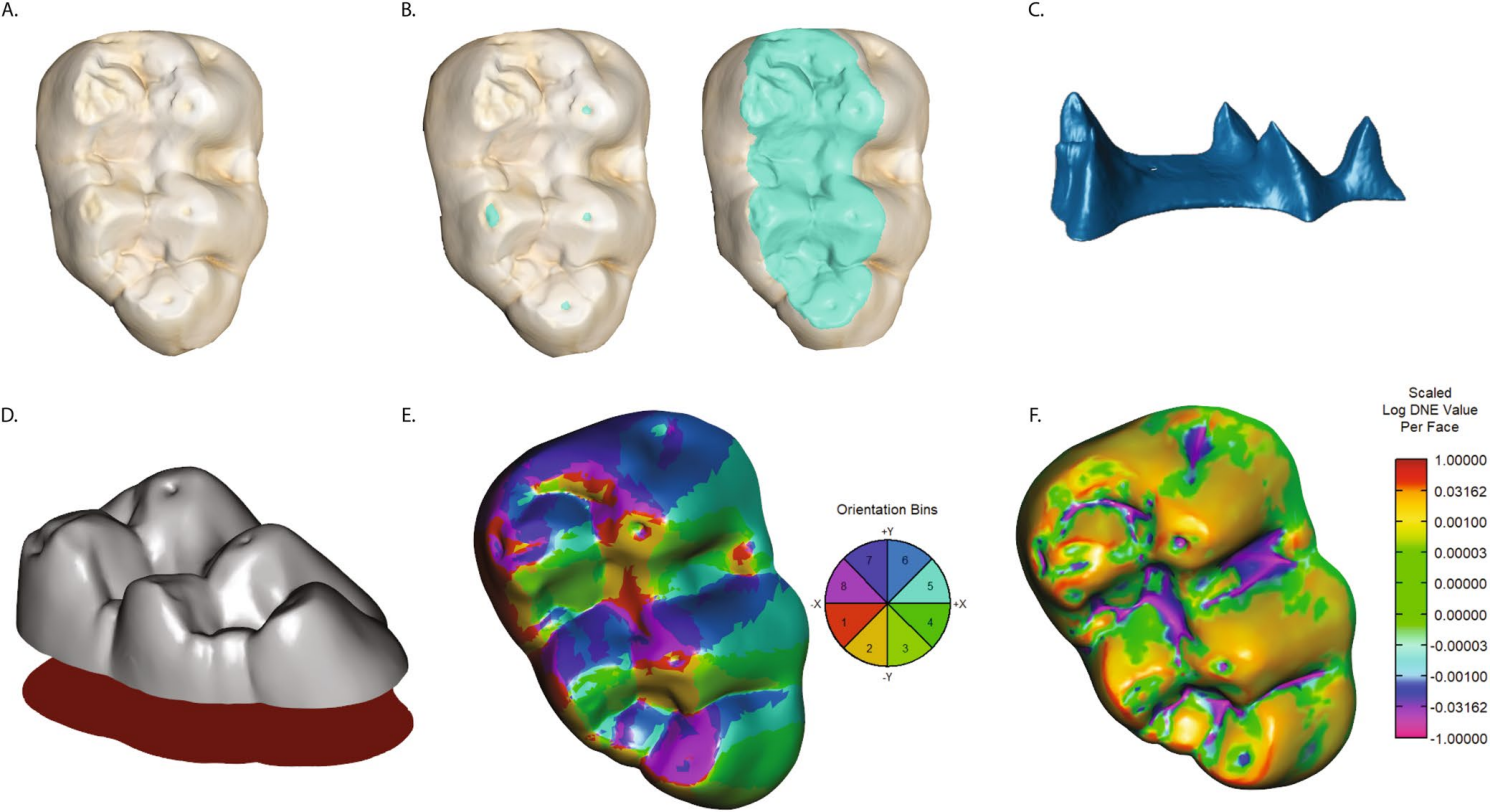
Data collected for a single right *M*_3_ from SNPRC animal number 174: (A) 3D scan, (B) DER ratio, (C) extracted pulp volume used to calculate CPV, (D) RFI, (E) OPCR, and (F) vDNE.

### 2.2 MicroCT Scanning

All 46 baboon mandibles were scanned using a Bruker SkyScan 1273 micro‐computed tomography system at Loyola University Chicago. Scans were acquired at an isotropic voxel size of 35–39 μm using an aluminum‐copper (Al 1 mm‐Cu 0.038 mm) filter and large focal spot. This small variation in voxel size is unlikely to meaningfully affect volumetric or topographic measurements. Specimen‐specific voxel sizes are provided in Supplementary Online Material (SOM Table 1). All scans were acquired at a voltage of 100 kV, a current of 300 μA, a 330 ms exposure, a 0.15**°** rotation step, and a 360**°** rotation range. Projection images were reconstructed using NRecon software (Bruker microCT) with smoothing set to two, ring artifact reduction set to five, and beam‐hardening correction set to 15%. The reconstructed image stacks were exported as TIFF files for segmentation and volumetric analyses.

Pulp chamber volume was measured from the reconstructed microCT datasets in Dragonfly (Object Research Systems, Montreal, Canada), following Selig et al. (2025). For each molar, the pulp chamber was segmented from the surrounding dentin using grayscale thresholding with manual refinement in orthogonal slice views. Segmentation was restricted to the coronal portion of the pulp chamber by cropping each dataset along a plane parallel to the floor of the pulp chamber at the level of the pulp bridge. The segmented pulp chamber was rendered as a three‐dimensional object and total pulp chamber volume was calculated using Dragonfly's volumetric measurement tools (Figures 1 and 2).

**FIGURE 2:**
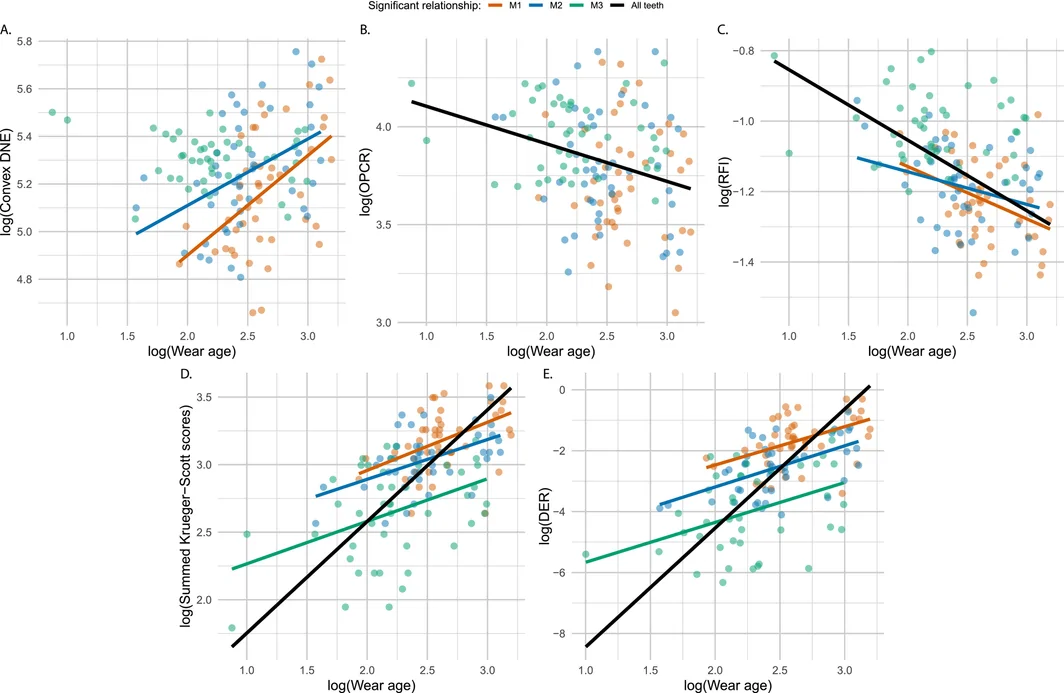
Relationships between wear age and dental topography measures (A–C), summed Krueger‐Scott scores (D), and DER (E). All variables are natural log‐transformed. The black lines indicate significant relationships across all teeth (*p* < 0.05), orange for *M*_1_, blue for *M*_2_, and green for *M*_3_.

For dental topographic analyses, the crown of each tooth was segmented by setting a cropping plane parallel to the enamel cervix and cropping each dataset above the pulp cavity horns. Crown meshes were simplified to 10,000 faces, smoothed for 10 iterations with a lambda of 0.5 in vcgSmooth (taubin), and exported as PLY files (Morley and Berthaume 2023; Pampush, Winchester, et al. 2016; Spradley et al. 2017). Dental topography metrics were calculated using these PLY files in the R package MolaR (Pampush, Winchester, et al. 2016). Three measures of dental topography were calculated (Figure 1). The relief index (RFI) represents the ratio of crown surface area to its two‐dimensional occlusal footprint (Boyer 2008; Pampush, Winchester, et al. 2016; Ungar and M'Kirera 2003). Orientation patch count rotated (OPCR) was calculated by dividing the occlusal surface into eight compass directions and has been used as a proxy for the number of potential shearing sites on the occlusal surface (Evans et al. 2007). While this measure has proven effective for lophodont taxa (Evans et al. 2007; Evans and Janis 2014), the relationship between OPCR and dietary fiber content is less robust in taxa with tribosphenic and bunodont dentition (Pineda‐Munoz et al. 2017; Winchester et al. 2014). Finally, convex Dirichlet normal energy (vDNE) was used to quantify curvature across the occlusal surface and reflects the sharpness of occlusal features associated with functional tooth contact (Pampush, Winchester, et al. 2016; Pampush et al. 2022).

The study sample of SNPRC baboons used herein is part of a collection of approximately 1000 skulls that were CT‐scanned in the early 2000s (Sherwood et al. 2008; Roseman et al. 2010; Joganic et al. 2018). These earlier scans are on MorphoSource and have continued to be used for quantitative genetic analyses of cranial variation (e.g., Thiebaut et al. 2026). For readers who want to utilize these older shared resources, we note that some metadata details for these early scans need to be adjusted prior to use (Modesto‐Mata et al. 2025).

### 2.3 Analyses

As baboon molars differ in size, we calculated a corrected pulp volume (CPV) defined as the pulp chamber volume divided by two‐dimensional occlusal area × 100 (Selig et al. 2025). To test relationships between dental wear and age, we accounted for differences in molar eruption timing. Captive baboons at the SNPRC have earlier dental eruption times compared to wild baboons (Phillips‐Conroy and Jolly 1988). Therefore, sex‐specific average eruption ages for this colony from Phillips‐Conroy and Jolly (1988) were subtracted from the chronological age at death for each tooth. This produced a variable termed “wear age,” defined as the number of years each tooth had been in occlusion. All tested variables were significantly heteroskedastic (Breusch–Pagan test, *p* < 0.01) and were natural log‐transformed prior to any analyses.

Linear mixed effects models were implemented in the R package “lme4” to test relationships between dental topography or wear variables against wear age and CPV across all teeth. Because multiple teeth were sampled from the same individuals, animal identification was included as a random factor in all analyses. Sex was also added as an explanatory factor in all models. Model fit was evaluated using the “performance” package, which reports marginal (*r* ^2^ *m*) and conditional (*r* ^2^ *c*) coefficients of determination representing the variance explained by fixed effects alone and by both fixed and random effects, respectively. Slope differences were tested using pairwise comparisons. Models were first run across all pooled molars testing relationships between wear age and wear and topography variables (Q1) and between wear and topography variables and CPV (Q2). To compare these relationships between molars, likelihood‐ratio tests were first used to test whether the explanatory variable improved the model beyond tooth position alone. For relationships improved by tooth position, tooth position was then included in all models as an interaction term. To test the relative contributions of each wear and topography variable on CPV, a linear mixed effects model with all variables was fit including animal as a random factor. The contribution of each factor was quantified using a semi‐partial marginal *R* ^2^ in the R package “partR2” over 1000 iterations (Stoffel et al. 2021).

## 3 Results

### 3.1 Q1. How Do Measures of Dental Topography and Dental Wear Vary With Wear Age in Baboons?

Measures of dental topography varied significantly across the molar row. Specifically, vDNE and OPCR were significantly higher on *M*_3_ compared to *M*_1_, whereas RFI was significantly lower on *M*_3_ compared to *M*_1_ and *M*_2_ (SOM Table 2). Summed Krueger‐Scott scores, vDNE, and wear age also varied across the toothrow with M_3_ having significantly lower values than M_2_ and M_1_, consistent with expectations based on eruption order (SOM Table 3). Wear and dental topography variables were also tested for differences between males and females. Males had significantly larger OPCR and summed Krueger‐Scott scores than females (SOM Table 3).

Across the pooled molar sample, summed Krueger‐Scott scores and DER increased significantly with wear age (Figure 2; Table 2; SOM Table 4; *r* ^2^ *c* = 0.84–0.87; *r* ^2^ *m* = 0.53–0.55; *p* < 0.01). Among the dental topography variables, OPCR showed a significant negative correlation with wear age across all molars (*r* ^2^ *c* = 0.24; *r* ^2^ *m* = 0.08; *p* < 0.01), as did RFI (*r* ^2^ *c* = 0.52; *r* ^2^ *m* = 0.29; *p* < 0.01). Convex DNE was not significantly associated with wear age across all molars (*p* = 0.19).

**TABLE 2:** Summary of Q1 relationships between wear age and measures of dental wear and dental topography (full results in SOM Table 4; KS scores‐summed Krueger‐Scott scores; *R* ^2^ *m*‐marginal *r* ^2^; *R* ^2^ *c*‐conditional *r* ^2^).

Response (log)	Model	*n*	Estimate and *p*‐value	*R* ^2^ *m*	*R* ^2^ *c*
DER	Pooled	130	3.91; *p* < 0.001	0.55	0.87
DER	Tooth	130	M1: 1.26, *p* = < 0.01; M2: 1.35, *p* = < 0.001; M3: 1.31, *p* = < 0.001	0.57	0.83
KS scores	Pooled	134	0.83; < 0.001	0.53	0.84
KS scores	Tooth	134	M1: 0.36, *p* = < 0.01; M2: 0.29, *p* = < 0.01; M3: 0.32, *p* = < 0.001	0.52	0.81
OPCR	Pooled	136	−0.19; < 0.01	0.08	0.23
OPCR	Tooth	136	M1: −0.09, *p* = 0.49; M2: −0.13, *p* = 0.23; M3: −0.04, *p* = 0.66‐Model not supported	0.14	0.33
RFI	Pooled	136	−0.2; < 0.001	0.29	0.52
RFI	Tooth	136	M1: −0.15, *p* = < 0.01; M2: −0.09, *p* = 0.04; M3: −0.03, *p* = 0.43	0.44	0.71
vDNE	Pooled	136	−0.07; 0.19	0.02	0.42
vDNE	Tooth	136	M1: 0.42, *p* = < 0.001; M2: 0.28, *p* = < 0.001; M3: −0.04, *p* = 0.55	0.26	0.55

Likelihood‐ratio tests indicated that wear age explained significantly more variation in vDNE, DER, RFI, and Krueger‐Scott score models compared to tooth position alone, but not OPCR (SOM Table 5). When tooth position was included as an interaction term, summed Krueger‐Scott scores and DER remained significantly positively correlated with wear age for each molar. The intercepts of these relationships were highest for M_1_ and lowest for M_3_ (Figure 2; Table 2; SOM Table 4; all *p* < 0.01), although the slopes of these relationships did not differ significantly across the molars (SOM Table 6). As expected, OPCR was not significantly associated with wear age for individual molars. RFI was significantly negatively correlated with wear age in *M*_1_ and *M*_2_, and vDNE was positively correlated with wear age in *M*_1_ and *M*_2_. The only relationships with significant differences in slope were between *M*_3_ and *M*_1_ and *M*_2_ for vDNE and wear age, and between *M*_1_ and *M*_3_ for RFI and wear age (SOM Table 6).

### 3.2 Q2. Is Pulp Chamber Volume More Strongly Associated With Cumulative Dental Wear or With Changes in Dental Topography?

Across all teeth, corrected pulp volume (CPV) decreased significantly with wear age, summed Krueger‐Scott scores, and DER (Figure 3; Table 3; all *p* < 0.01; SOM Table 7). All models showed similar conditional *R* ^2^ values in CPV, with Krueger‐Scott scores at 0.47, DER at 0.50, and wear age at 0.49. Among the dental topography variables, only RFI was significantly associated with CPV across all molars (Figure 3; *r* ^2^ *c* = 0.38; *r* ^2^ *m* = 0.03; *p* = 0.05).

**FIGURE 3:**
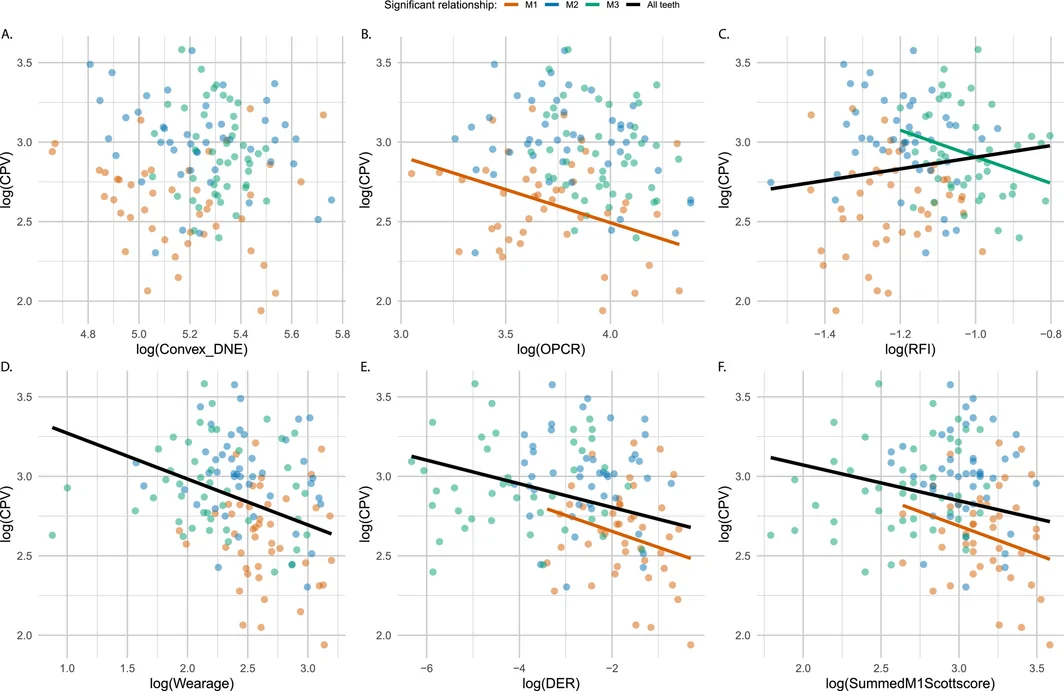
Relationships between CPV and dental topography measures (A–C), wear age (D), DER (E), and summed Krueger‐Scott scores (F). All variables are natural log‐transformed. The black lines indicate significant (*p* < 0.05) relationships across all teeth, orange for *M*_1_, blue for *M*_2_, and green for *M*_3_.

**TABLE 3:** Summary of Q2 relationships between CPV and measures of dental wear and dental topography (full results in SOM Table 7; KS scores‐summed Krueger‐Scott scores; *R* ^2^ *m*‐marginal *r* ^2^; *R* ^2^ *c*‐conditional *r* ^2^).

Response (log)	Model	*n*	Estimate and *p*‐value	*R* ^2^ *m*	*R* ^2^ *c*
Wear age	Pooled	135	−0.29; *p* = < 0.001	0.12	0.49
Wear age	Tooth	135	M1: −0.11, *p* = 0.44; M2: −0.15, *p* = 0.20; M3: −0.04, *p* = 0.62‐Model not supported	0.28	0.72
vDNE	Pooled	135	0.04; *p* = 0.75	0	0.33
vDNE	Tooth	135	M1: −0.26, *p* = 0.05; M2: −0.19, *p* = 0.19; M3: −0.19, *p* = 0.51‐Model not supported	0.28	0.73
OPCR	Pooled	135	−0.02; *p* = 0.81	0	0.32
OPCR	Tooth	135	M1: −0.42, *p* = < 0.001; M2: −0.16, *p* = 0.14; M3: −0.05, *p* = 0.76	0.3	0.78
RFI	Pooled	135	0.37; *p* = 0.05	0.03	0.38
RFI	Tooth	135	M1: 0.34, *p* = 0.22; M2: −0.29, *p* = 0.29; M3: −0.83, *p* = < 0.01	0.29	0.76
KS scores	Pooled	133	−0.23; *p* = < 0.01	0.06	0.47
KS scores	Tooth	133	M1: −0.36, *p* = 0.01; M2: 0.01, *p* = 0.96; M3: 0.15, *p* = 0.10	0.27	0.8
DER	Pooled	129	−0.07; *p* = < 0.001	0.1	0.5
DER	Tooth	129	M1: −0.10, *p* = 0.02; M2: 0.01, *p* = 0.71; M3: 0.03, *p* = 0.24	0.27	0.8

Likelihood‐ratio tests indicate that wear age and vDNE did not explain additional variation in CPV compared to tooth position alone (SOM Table 8). When tooth position was included as an interaction term, *M*_1_ DER and summed Krueger‐Scott scores were negatively correlated with CPV (Figure 3; SOM Table 7). Among dental topography variables, *M*_1_ OPCR values were negatively correlated with CPV. Finally, *M*_3_ RFI values were negatively correlated with CPV. Slope tests were not run because no relationship had more than one tooth with a significant relationship to CPV. A multivariate model testing the contributions of each wear and topography variable indicated that wear age followed by DER explained the greatest amount of variation in CPV (SOM Table 9).

## 4 Discussion

Although dental wear accumulates across an individual's lifespan, teeth exhibit a range of proactive and reactive adaptations that mitigate the functional consequences of wear. One such adaptation is dentin deposition within the pulp chamber, which reduces pulp volume over time. Here we evaluated this response in a sample of baboons with known ages of death by examining relationships between pulp chamber volume, dental wear, and dental topography. Although our sample represents the largest comparison of CPV in primates to date, some analyses remain limited by sample size. Individual‐level ancestry estimates were not available for these specimens, preventing us from incorporating hybrid composition as a covariate. Hybrid composition therefore remains a limitation that should be evaluated in future samples with individual‐level ancestry data. Together, these findings indicate that pulp chamber reduction in baboons reflects both cumulative wear and occlusal morphology. Among the dental topography variables, RFI was the only measure associated with CPV across the pooled molar sample.

### 4.1 Wear Age Shows Few Relationships With Dental Topography

As expected for our sample, wear age was positively related to summed Krueger–Scott scores and DER, indicating that both ordinal scoring and quantitative measures of exposed dentin capture broad age‐graded wear progression. Wear age conditionally captured similar amounts of variation in summed Krueger–Scott scores and DER (84%–87%), although some of the teeth in our sample had worn enamel but little to no dentin exposure. We also observed expected differences in dental wear across the molar row. Distal molars exhibited relatively less wear compared to the mesial molars, which reflects differences in eruption timing, and relationships between wear age and topographic variables are weaker for the *M*_3_ *s*.

Dental topographic approaches were developed in part to circumvent the “worn tooth conundrum” by quantifying surface form without requiring homologous landmarks that are progressively removed by wear (Ungar and M'Kirera 2003; Ungar and Williamson 2000). Subsequent work demonstrates that topographic wear trajectories can differ across primate taxa and dental morphologies, with some species showing strong, directional change in measures such as relief and curvature as wear progresses (e.g., Berthaume et al. 2019; Pampush, Spradley, et al. 2016; Pampush et al. 2018; Winchester et al. 2014). In Papionini, for example, dietary signals in dental topography can persist across varying degrees of wear (Avià et al. 2022). However, our results suggest that baboon molars show relatively weak relationships between wear age and most measures of dental topography. Higher wear ages were associated with lower RFI across all teeth and within *M*_1_ and *M*_2_ (Table 2), meaning that the teeth became flatter with age. OPCR showed a similar relationship across all teeth in that the complexity of the occlusal surface decreased at higher ages, but wear age only captured 23% of the conditional variation in OPCR, and there were no molar‐specific correlations. The weak relationship between OPCR and wear age is consistent with previous work suggesting that OPCR is most effective for distinguishing broad dietary differences among taxa rather than tracking within‐taxon wear trajectories (Pampush, Spradley, et al. 2016; Pineda‐Munoz et al. 2017). Convex DNE increased with wear age in *M*_1_ and *M*_2_, but not *M*_3_ or across all teeth (Figure 2). This pattern is consistent with the idea that wear can increase local surface curvature by creating sharper enamel–dentin transitions, facets, and surface complexity as cusps are progressively blunted. The absence of this relationship in *M*_3_ likely reflects its shorter duration in occlusion and lower overall wear. Together, these results indicate that while external measures of wear track cumulative tooth use, internal responses within the pulp chamber may be shaped not only by cumulative use of the tooth, but also by how occlusal structure mediates loading.

### 4.2 RFI Is the Only Topographic Measure Associated With CPV in Baboons

Wear age, DER, and summed Krueger‐Scott scores were all negatively associated with CPV across all teeth, indicating that cumulative wear contributes to pulp chamber reduction in baboons, as expected. We also found that CPV across all pooled teeth was moderately predicted by RFI. While RFI in worn teeth is not independent of dental wear, this pattern suggests that pulp chamber reduction is also influenced by occlusal morphology. This interpretation is important because CPV cannot distinguish between age‐related deposition of secondary dentin and localized tertiary dentin formation in response to mechanical or other localized stimuli. Our results show that reductions in pulp chamber volume in this sample likely reflect both processes—a background trend of age‐related pulp reduction and a super‐imposed response to loading‐related stimuli within the pulp‐dentin complex.

These differences suggest taxon‐specific differences in pulpal stimulation and dental structure. We hypothesize that the association between RFI and CPV arises because aspects of occlusal geometry captured by RFI, including relative cusp height and relief, may affect how forces are distributed across the crown. RFI in worn teeth reflects a combination of dental wear and functional signals, and wear‐related changes in occlusal relief and surface geometry may alter how loads are distributed across the crown. Tertiary dentin deposition can be triggered by localized, often episodic stimuli to the pulp‐dentin complex rather than by enamel loss alone (Ricucci et al. 2014). We did not have direct measures of masticatory loading, enamel thickness or volume, initial pulp dimensions, or histological dentin type for these individuals. Because baboons tend to accumulate dental wear on their molar cusps, relative cusp height may buffer the transmission of stress to the pulp–dentin complex. Occlusal relief is also hypothesized to influence how masticatory forces are transmitted through the tooth crown. While loading data are not available for these baboons, teeth with higher cusps may concentrate occlusal loads along crests and facets, potentially producing localized stress within the pulp‐dentin complex. Under this interpretation, tertiary dentin deposition may reflect patterns of localized occlusal loading rather than cumulative enamel loss alone (Goel et al. 1990; Nudel et al. 2021). It is also worth noting that CPV is a whole‐tooth measure, whereas wear and dental topography capture surface‐level and often localized changes. If dentin deposition occurs beneath specific areas of concentrated loading, the whole‐tooth measure of pulp chamber volume may dilute these localized responses.

Variation in enamel structure may further modulate this relationship, although the precise role of baboon enamel thickness remains unresolved. Absolute enamel thickness varies within baboons (Hlusko et al. 2004), potentially a function of latitudinal variation within baboon species (Kato et al., forthcoming). Evidence for metameric variation in baboon molar enamel thickness is mixed (Hlusko et al. 2004; Grine et al. 2005), but recent work suggests that enamel structural properties such as enamel prism dimensions may also differ from other primates (Towle and Loch 2024). Considering the high degree of enamel thickness variation, thinner enamel may allow dentin exposure to stimulate pulp responses earlier during the wear process, whereas thicker enamel may buffer the pulp‐dentin complex from similar stimulation despite comparable levels of occlusal wear. This variation may itself be responsive to dietary selective pressures (Hlusko et al. 2004), resulting in taxon‐specific differences in pulpal stimulation and dental structure.

Our sample of baboons was derived from a captive population. While there were teeth with extensive wear, the degree of wear was likely lower than expected in a wild population. Wild baboons frequently consume foods that are both mechanically challenging and contaminated with environmental grit (Fannin et al. 2021), and rates of dental wear in wild populations are thought to reflect the combined effects of exogenous abrasives and the mechanical properties of consumed foods (Galbany et al. 2014). In contrast, the captive baboons in this study consumed primarily monkey chow supplemented with enrichment foods (and some individuals preferred to soak their monkey chow pellets in water before consuming, L. Hlusko pers. comm.), which likely produced lower levels of abrasive wear and reduced mechanical loading compared to wild populations. As a result, the pulp‐dentin complex in these animals may have experienced fewer stimuli capable of triggering tertiary dentin deposition.

Taken together, these observations suggest that the relationship between CPV and dental wear may be highly sensitive to occlusal relief, dietary mechanics, and loading regimes. In particular, loading environments that produce concentrated forces and localized dentin stimuli may be most likely to trigger tertiary dentin deposition and subsequent reductions in pulp chamber volume (Towle et al. 2026). This interpretation highlights the importance of considering both dietary mechanics and environmental abrasives when evaluating internal dental responses to wear in primates.

## 5 Conclusions

Our results suggest that pulp chamber reduction in baboons reflects both cumulative wear and the functional effects of occlusal morphology, particularly occlusal relief. These findings indicate that comparisons of CPV across taxa and dietary categories may be complicated by structural differences in tooth form as well as differences in loading environment. More broadly, internal tooth responses to wear may be buffered and patterned by crown morphology, meaning that pulp chamber remodeling may track not only cumulative enamel loss but also how wear is mechanically experienced by the tooth.

## Author Contributions

**Myra F. Laird:** conceptualization, investigation, writing – original draft, methodology, writing – review and editing, formal analysis, project administration, supervision, resources. **Diego A. Contreras:** investigation, methodology, validation, visualization, writing – review and editing. **Leslea J. Hlusko:** writing – review and editing, investigation, project administration, data curation, resources. **Kristin L. Krueger:** conceptualization, writing – review and editing, investigation, methodology, visualization, validation, project administration, resources, data curation.

## Funding

This work was supported by the National Science Foundation (2418553, 2418554), Penn Dental Medicine's Rabinowitz Award, Loyola University Research Support Grant (1235), and HORIZON EUROPE European Research Council (101054659).

## Supporting information


**SOM Table 1.** MicroCT acquisition parameters for all specimens scanned using a Bruker SkyScan 1273. Reconstruction parameters (smooth—2, ring artifact correction = 5, beam hardening correction = 15%) were held constant across all scans.
**SOM Table 2.** Differences in tested variables between the molars.
**SOM Table 3.** Sex differences in the tested wear and topography variables.
**SOM Table 4.** Relationships between wear age and measures of dental wear and dental topography. *R* ^2^ *m*‐marginal *r* ^2^, *R* ^2^ *c*‐conditional *r* ^2^.
**SOM Table 5.** Likelihood‐ratio test results comparing tooth‐only models with models including an interaction between each predictor and tooth.
**SOM Table 6.** Table of slope differences between molars for wear and topography measures against wear age.
**SOM Table 7.** Relationships between CPV and measures of dental wear and dental topography. *R* ^2^ *m*‐marginal *r* ^2^, *R* ^2^ *c*‐conditional *r* ^2^.
**SOM Table 8.** Likelihood‐ratio test results comparing tooth‐only models with models including an interaction between each predictor and tooth.
**SOM Table 9.** Semi‐partial marginal *R* ^2^ values and confidence intervals for the contributions of each variable to CPV in a multivariate model.

## Data Availability

All derived data are available on Dryad: https://doi.org/10.5061/dryad.ns1rn8q8f.
