# Supplementary material for: Occlusal Relief and Dental Wear Predict Pulp Chamber Volume in Captive Baboons
Source: Am J Biol Anthropol. 2026 Oct 30;191(3):e70375. doi: 10.1002/ajpa.70375 (PMC13722879; doi:10.1002/ajpa.70375)
Supplement: Supplementary file 1 [file AJPA-191-e70375-s001.docx]

**Occlusal relief and dental wear predict pulp chamber volume in captive baboons**

Myra F. Laird^1*^, Diego A. Contreras^1,2^, Leslea J. Hlusko³, Kristin L. Krueger^4^

^1^Department of Basic and Translational Sciences, University of Pennsylvania

²Department of Anthropology, McGill University

³National Research Center on Human Evolution (CENIEH)

^4^Department of Anthropology, Loyola University Chicago

*mflaird@upenn.edu

**Supplemental Online Material**

**SOM Table 1.** MicroCT acquisition parameters for all specimens scanned using a Bruker SkyScan 1273. Reconstruction parameters (smooth - 2, ring artifact correction = 5, beam hardening correction = 15%) were held constant across all scans.

| **SNPRC animal number** | **Voxel size (µm)** |
| --- | --- |
| 23 | 37 |
| 39 | 39 |
| 48 | 39 |
| 49 | 37 |
| 87 | 39 |
| 163 | 39 |
| 172 | 37 |
| 174 | 39 |
| 178 | 37 |
| 189 | 38 |
| 190 | 37 |
| 196 | 39 |
| 201 | 39 |
| 224 | 39 |
| 231 | 37 |
| 253 | 37 |
| 259 | 37 |
| 260 | 37 |
| 265 | 37 |
| 281 | 37 |
| 288 | 37 |
| 311 | 37 |
| 312 | 37 |
| 316 | 39 |
| 317 | 37 |
| 322 | 37 |
| 330 | 36 |
| 332 | 37 |
| 361 | 37 |
| 378 | 37 |
| 382 | 37 |
| 384 | 37 |
| 387 | 37 |
| 393 | 37 |
| 394 | 37 |
| 398 | 37 |
| 400 | 39 |
| 401 | 37 |
| 405 | 37 |
| 406 | 39 |
| 412 | 37 |
| 421 | 39 |
| 424 | 37 |
| 432 | 39 |
| 443 | 37 |
| 456 | 35 |

**SOM Table 2**. Differences in tested variables between the molars.

| **Variable** | **contrast** | **n** | **estimate** | **SE** | **df** | **t** | **p.adj** |
| --- | --- | --- | --- | --- | --- | --- | --- |
| OPCR | M2 - M1 | 136 | 0.08 | 0.05 | 88.57 | 1.70 | 0.25 |
| OPCR | M3 - M1 | 136 | 0.23 | 0.05 | 89.08 | 4.92 | <0.001 |
| OPCR | M3 - M2 | 136 | 0.15 | 0.05 | 88.57 | 3.25 | <0.001 |
| RFI | M2 - M1 | 136 | 0.01 | 0.00 | 88.30 | 2.16 | 0.10 |
| RFI | M3 - M1 | 136 | 0.05 | 0.00 | 88.59 | 12.49 | <0.001 |
| RFI | M3 - M2 | 136 | 0.04 | 0.00 | 88.30 | 10.43 | <0.001 |
| vDNE | M2 - M1 | 136 | 0.07 | 0.03 | 88.40 | 2.14 | 0.10 |
| vDNE | M3 - M1 | 136 | 0.14 | 0.03 | 88.77 | 4.10 | <0.001 |
| vDNE | M3 - M2 | 136 | 0.07 | 0.03 | 88.40 | 1.99 | 0.14 |
| Summed Krueger-Scott Scores | M2 - M1 | 134 | -0.15 | 0.03 | 86.22 | -4.87 | <0.001 |
| Summed Krueger-Scott Scores | M3 - M1 | 134 | -0.51 | 0.03 | 86.88 | -16.31 | <0.001 |
| Summed Krueger-Scott Scores | M3 - M2 | 134 | -0.36 | 0.03 | 86.71 | -11.57 | <0.001 |
| Wear age | M2 - M1 | 136 | -0.16 | 0.01 | 88.02 | -10.91 | <0.001 |
| Wear age | M3 - M1 | 136 | -0.38 | 0.01 | 88.04 | -26.08 | <0.001 |
| Wear age | M3 - M2 | 136 | -0.22 | 0.01 | 88.02 | -15.39 | <0.001 |
| DER | M2 - M1 | 130 | -0.11 | 0.01 | 82.31 | -8.07 | <0.001 |
| DER | M3 - M1 | 130 | -0.17 | 0.01 | 84.11 | -12.41 | <0.001 |
| DER | M3 - M2 | 130 | -0.07 | 0.01 | 83.85 | -4.69 | <0.001 |

**SOM Table 3**. Sex differences in the tested wear and topography variables.

| **Variable** | **Total n** | **estimate** | **std_error** | **df** | **t** | **P value** |
| --- | --- | --- | --- | --- | --- | --- |
| CPV | 135 | 0.01 | 0.08 | 45.33 | 0.19 | 0.85 |
| vDNE | 136 | 0.04 | 0.05 | 43.57 | 0.77 | 0.45 |
| DER | 130 | 0.04 | 0.03 | 42.91 | 1.45 | 0.15 |
| OPCR | 136 | 0.17 | 0.05 | 42.83 | 3.48 | <0.001 |
| RFI | 136 | -0.01 | 0.01 | 44.80 | -1.91 | 0.06 |
| Summed Krueger-Scott score | 134 | 0.20 | 0.07 | 45.49 | 2.96 | <0.001 |
| Wear age | 136 | -0.08 | 0.11 | 44.01 | -0.70 | 0.49 |

**SOM Table 4**. Relationships between wear age and measures of dental wear and dental topography. R2m-marginal r^2^, R2c-conditional r^2^.

| **model** | **n** | **term** | **estimate** | **std.error** | **statistic** | **df** | **p.value** | **R2m** | **R2c** |
| --- | --- | --- | --- | --- | --- | --- | --- | --- | --- |
| log(vDNE) ~ log(Wear age) (pooled) | 136 | (Intercept) | 5.41 | 0.13 | 42.13 | 84.16 | <0.001 | 0.02 | 0.42 |
| log(vDNE) ~ log(Wear age) (pooled) | 136 | x | -0.07 | 0.05 | -1.31 | 89.28 | 0.19 | 0.02 | 0.42 |
| log(vDNE) ~ log(Wear age)*Tooth | 136 | (Intercept) | 4.06 | 0.25 | 16.58 | 107.14 | <0.001 | 0.26 | 0.55 |
| log(vDNE) ~ log(Wear age)*Tooth | 136 | x | 0.42 | 0.09 | 4.55 | 107.64 | <0.001 | 0.26 | 0.55 |
| log(vDNE) ~ log(Wear age)*Tooth | 136 | ToothM2 | 0.49 | 0.25 | 1.99 | 94.84 | 0.05 | 0.26 | 0.55 |
| log(vDNE) ~ log(Wear age)*Tooth | 136 | ToothM3 | 1.33 | 0.23 | 5.78 | 115.63 | <0.001 | 0.26 | 0.55 |
| log(vDNE) ~ log(Wear age)*Tooth | 136 | x:ToothM2 | -0.14 | 0.09 | -1.48 | 92.78 | 0.14 | 0.26 | 0.55 |
| log(vDNE) ~ log(Wear age)*Tooth | 136 | x:ToothM3 | -0.46 | 0.09 | -5.16 | 105.64 | <0.001 | 0.26 | 0.55 |
| log(DER) ~ log(Wear age) (pooled) | 130 | (Intercept) | -12.35 | 0.78 | -15.92 | 128.91 | <0.001 | 0.55 | 0.87 |
| log(DER) ~ log(Wear age) (pooled) | 130 | x | 3.91 | 0.31 | 12.82 | 129.84 | <0.001 | 0.55 | 0.87 |
| log(DER) ~ log(Wear age)*Tooth | 130 | (Intercept) | -4.99 | 1.23 | -4.05 | 82.17 | <0.001 | 0.57 | 0.83 |
| log(DER) ~ log(Wear age)*Tooth | 130 | x | 1.26 | 0.46 | 2.72 | 82.93 | 0.01 | 0.57 | 0.83 |
| log(DER) ~ log(Wear age)*Tooth | 130 | ToothM2 | -0.90 | 1.02 | -0.89 | 92.79 | 0.38 | 0.57 | 0.83 |
| log(DER) ~ log(Wear age)*Tooth | 130 | ToothM3 | -1.98 | 1.03 | -1.92 | 118.94 | 0.06 | 0.57 | 0.83 |
| log(DER) ~ log(Wear age)*Tooth | 130 | x:ToothM2 | 0.09 | 0.39 | 0.24 | 89.14 | 0.81 | 0.57 | 0.83 |
| log(DER) ~ log(Wear age)*Tooth | 130 | x:ToothM3 | 0.05 | 0.39 | 0.12 | 106.46 | 0.90 | 0.57 | 0.83 |
| log(OPCR) ~ log(Wear age) (pooled) | 136 | (Intercept) | 4.30 | 0.15 | 28.95 | 74.87 | <0.001 | 0.08 | 0.24 |
| log(OPCR) ~ log(Wear age) (pooled) | 136 | x | -0.19 | 0.06 | -3.23 | 77.01 | <0.001 | 0.08 | 0.24 |
| log(OPCR) ~ log(Wear age)*Tooth | 136 | (Intercept) | 3.96 | 0.34 | 11.67 | 125.63 | <0.001 | 0.14 | 0.33 |
| log(OPCR) ~ log(Wear age)*Tooth | 136 | x | -0.09 | 0.13 | -0.71 | 125.89 | 0.48 | 0.14 | 0.33 |
| log(OPCR) ~ log(Wear age)*Tooth | 136 | ToothM2 | 0.16 | 0.38 | 0.43 | 91.92 | 0.67 | 0.14 | 0.33 |
| log(OPCR) ~ log(Wear age)*Tooth | 136 | ToothM3 | 0.08 | 0.35 | 0.23 | 106.59 | 0.82 | 0.14 | 0.33 |
| log(OPCR) ~ log(Wear age)*Tooth | 136 | x:ToothM2 | -0.04 | 0.15 | -0.26 | 90.69 | 0.79 | 0.14 | 0.33 |
| log(OPCR) ~ log(Wear age)*Tooth | 136 | x:ToothM3 | 0.05 | 0.14 | 0.40 | 99.66 | 0.69 | 0.14 | 0.33 |
| log(RFI) ~ log(Wear age) (pooled) | 136 | (Intercept) | -0.65 | 0.08 | -8.66 | 82.42 | <0.001 | 0.29 | 0.52 |
| log(RFI) ~ log(Wear age) (pooled) | 136 | x | -0.20 | 0.03 | -6.63 | 85.91 | <0.001 | 0.29 | 0.52 |
| log(RFI) ~ log(Wear age)*Tooth | 136 | (Intercept) | -0.83 | 0.14 | -5.96 | 98.97 | <0.001 | 0.45 | 0.71 |
| log(RFI) ~ log(Wear age)*Tooth | 136 | x | -0.15 | 0.05 | -2.83 | 99.50 | 0.01 | 0.45 | 0.71 |
| log(RFI) ~ log(Wear age)*Tooth | 136 | ToothM2 | -0.13 | 0.13 | -0.96 | 96.65 | 0.34 | 0.45 | 0.71 |
| log(RFI) ~ log(Wear age)*Tooth | 136 | ToothM3 | -0.13 | 0.13 | -1.03 | 120.39 | 0.31 | 0.45 | 0.71 |
| log(RFI) ~ log(Wear age)*Tooth | 136 | x:ToothM2 | 0.06 | 0.05 | 1.11 | 94.10 | 0.27 | 0.45 | 0.71 |
| log(RFI) ~ log(Wear age)*Tooth | 136 | x:ToothM3 | 0.12 | 0.05 | 2.54 | 109.17 | 0.01 | 0.45 | 0.71 |
| log(Summed Krueger-Scott scores) ~ log(Wear age) (pooled) | 134 | (Intercept) | 0.93 | 0.18 | 5.27 | 128.26 | <0.001 | 0.53 | 0.84 |
| log(Summed Krueger-Scott scores) ~ log(Wear age) (pooled) | 134 | x | 0.83 | 0.07 | 11.89 | 132.75 | <0.001 | 0.53 | 0.84 |
| log(Summed Krueger-Scott scores) ~ log(Wear age)*Tooth | 134 | (Intercept) | 2.24 | 0.32 | 6.93 | 83.12 | <0.001 | 0.52 | 0.82 |
| log(Summed Krueger-Scott scores) ~ log(Wear age)*Tooth | 134 | x | 0.36 | 0.12 | 2.93 | 83.89 | <0.001 | 0.52 | 0.82 |
| log(Summed Krueger-Scott scores) ~ log(Wear age)*Tooth | 134 | ToothM2 | 0.06 | 0.27 | 0.22 | 97.04 | 0.83 | 0.52 | 0.82 |
| log(Summed Krueger-Scott scores) ~ log(Wear age)*Tooth | 134 | ToothM3 | -0.29 | 0.26 | -1.12 | 127.11 | 0.26 | 0.52 | 0.82 |
| log(Summed Krueger-Scott scores) ~ log(Wear age)*Tooth | 134 | x:ToothM2 | -0.06 | 0.10 | -0.61 | 93.19 | 0.54 | 0.52 | 0.82 |
| log(Summed Krueger-Scott scores) ~ log(Wear age)*Tooth | 134 | x:ToothM3 | -0.04 | 0.10 | -0.43 | 114.38 | 0.67 | 0.52 | 0.82 |
| log(vDNE) ~ log(Wear age)*Tooth | 136 | simple slope: M1 | 0.42 | 0.09 | 4.45 | 113.18 | <0.001 | NA | NA |
| log(vDNE) ~ log(Wear age)*Tooth | 136 | simple slope: M2 | 0.28 | 0.08 | 3.60 | 109.70 | <0.001 | NA | NA |
| log(vDNE) ~ log(Wear age)*Tooth | 136 | simple slope: M3 | -0.04 | 0.06 | -0.61 | 112.15 | 0.55 | NA | NA |
| log(DER) ~ log(Wear age)*Tooth | 130 | simple slope: M1 | 1.26 | 0.47 | 2.66 | 86.64 | 0.01 | NA | NA |
| log(DER) ~ log(Wear age)*Tooth | 130 | simple slope: M2 | 1.35 | 0.39 | 3.44 | 83.33 | <0.001 | NA | NA |
| log(DER) ~ log(Wear age)*Tooth | 130 | simple slope: M3 | 1.31 | 0.33 | 3.98 | 96.37 | <0.001 | NA | NA |
| log(OPCR) ~ log(Wear age)*Tooth | 136 | simple slope: M1 | -0.09 | 0.13 | -0.70 | 132.43 | 0.49 | NA | NA |
| log(OPCR) ~ log(Wear age)*Tooth | 136 | simple slope: M2 | -0.13 | 0.11 | -1.21 | 130.61 | 0.23 | NA | NA |
| log(OPCR) ~ log(Wear age)*Tooth | 136 | simple slope: M3 | -0.04 | 0.08 | -0.44 | 131.92 | 0.66 | NA | NA |
| log(RFI) ~ log(Wear age)*Tooth | 136 | simple slope: M1 | -0.15 | 0.05 | -2.77 | 103.90 | 0.01 | NA | NA |
| log(RFI) ~ log(Wear age)*Tooth | 136 | simple slope: M2 | -0.09 | 0.04 | -2.09 | 100.25 | 0.04 | NA | NA |
| log(RFI) ~ log(Wear age)*Tooth | 136 | simple slope: M3 | -0.03 | 0.03 | -0.80 | 102.81 | 0.43 | NA | NA |
| log(Summed Krueger-Scott scores) ~ log(Wear age)*Tooth | 134 | simple slope: M1 | 0.36 | 0.12 | 2.87 | 87.80 | 0.01 | NA | NA |
| log(Summed Krueger-Scott scores) ~ log(Wear age)*Tooth | 134 | simple slope: M2 | 0.30 | 0.10 | 2.86 | 84.46 | 0.01 | NA | NA |
| log(Summed Krueger-Scott scores) ~ log(Wear age)*Tooth | 134 | simple slope: M3 | 0.32 | 0.08 | 3.98 | 85.56 | <0.001 | NA | NA |

**SOM Table 5**. Likelihood-ratio test results comparing tooth-only models with models including an interaction between each predictor and tooth.

| **predictor** | **n** | **Chisq** | **p.value** |
| --- | --- | --- | --- |
| log(vDNE)~log(Wear Age) | 136 | 31.8 | <0.0001 |
| log(DER)~log(Wear Age) | 130 | 16.5 | 0.00091 |
| log(OPCR)~log(Wear Age) | 136 | 1.74 | 0.628 |
| log(RFI)~log(Wear Age) | 136 | 9.24 | 0.0263 |
| log(Summed Krueger-Scott scores)~log(Wear Age) | 134 | 15.3 | 0.00157 |

**SOM Table 6.** Table of slope differences between molars for wear and topography measures against wear age.

| Measure ~ Wear age | contrast | estimate_diff | SE | df | t_ratio | p_value |
| --- | --- | --- | --- | --- | --- | --- |
| log(vDNE) | M1 - M2 | 0.14 | 0.10 | 97.89 | 1.45 | 0.15 |
| log(vDNE) | M1 - M3 | 0.46 | 0.09 | 111.18 | 5.04 | <0.001 |
| log(vDNE) | M2 - M3 | 0.32 | 0.08 | 101.01 | 4.08 | <0.001 |
| log(DER) | M1 - M2 | -0.09 | 0.40 | 93.44 | -0.23 | 0.82 |
| log(DER) | M1 - M3 | -0.05 | 0.40 | 111.65 | -0.12 | 0.90 |
| log(DER) | M2 - M3 | 0.04 | 0.34 | 98.54 | 0.13 | 0.90 |
| log(OPCR) | M1 - M2 | 0.04 | 0.15 | 97.36 | 0.26 | 0.80 |
| log(OPCR) | M1 - M3 | -0.05 | 0.14 | 106.44 | -0.39 | 0.70 |
| log(OPCR) | M2 - M3 | -0.09 | 0.12 | 98.87 | -0.77 | 0.44 |
| log(RFI) | M1 - M2 | -0.06 | 0.05 | 98.33 | -1.08 | 0.28 |
| log(RFI) | M1 - M3 | -0.12 | 0.05 | 114.13 | -2.48 | 0.01 |
| log(RFI) | M2 - M3 | -0.07 | 0.04 | 102.38 | -1.57 | 0.12 |
| log(Summed Krueger-Scott scores) | M1 - M2 | 0.06 | 0.10 | 97.69 | 0.60 | 0.55 |
| log(Summed Krueger-Scott scores) | M1 - M3 | 0.04 | 0.10 | 119.85 | 0.42 | 0.68 |
| log(Summed Krueger-Scott scores) | M2 - M3 | -0.02 | 0.08 | 104.65 | -0.24 | 0.81 |

**SOM Table 7**. Relationships between CPV and measures of dental wear and dental topography. R2m-marginal r^2^, R2c-conditional r^2^.

| model | n | term | estimate | std.error | statistic | df | p.value | R2c | R2c |
| --- | --- | --- | --- | --- | --- | --- | --- | --- | --- |
| log(CPV) ~ log(Wear age) (pooled) | 135 | (Intercept) | 3.56 | 0.19 | 19.05 | 103.61 | <0.001 | 0.12 | 0.49 |
| log(CPV) ~ log(Wear age) (pooled) | 135 | x | -0.29 | 0.08 | -3.87 | 107.58 | <0.001 | 0.12 | 0.49 |
| log(CPV) ~ log(Wear age)*Tooth | 135 | (Intercept) | 2.91 | 0.37 | 7.87 | 84.55 | <0.001 | 0.28 | 0.72 |
| log(CPV) ~ log(Wear age)*Tooth | 135 | x | -0.11 | 0.14 | -0.80 | 85.41 | 0.43 | 0.28 | 0.72 |
| log(CPV) ~ log(Wear age)*Tooth | 135 | ToothM2 | 0.47 | 0.30 | 1.55 | 98.79 | 0.12 | 0.28 | 0.72 |
| log(CPV) ~ log(Wear age)*Tooth | 135 | ToothM3 | 0.12 | 0.30 | 0.40 | 127.79 | 0.69 | 0.28 | 0.72 |
| log(CPV) ~ log(Wear age)*Tooth | 135 | x:ToothM2 | -0.04 | 0.12 | -0.35 | 95.14 | 0.73 | 0.28 | 0.72 |
| log(CPV) ~ log(Wear age)*Tooth | 135 | x:ToothM3 | 0.07 | 0.11 | 0.59 | 114.76 | 0.56 | 0.28 | 0.72 |
| log(CPV) ~ log(Wear age)*Tooth simple slopes | 135 | simple slope: M1 | -0.11 | 0.14 | -0.78 | 89.23 | 0.44 | NA | NA |
| log(CPV) ~ log(Wear age)*Tooth simple slopes | 135 | simple slope: M2 | -0.15 | 0.12 | -1.30 | 83.93 | 0.20 | NA | NA |
| log(CPV) ~ log(Wear age)*Tooth simple slopes | 135 | simple slope: M3 | -0.05 | 0.09 | -0.50 | 86.09 | 0.62 | NA | NA |
| log(CPV) ~ log(vDNE) (pooled) | 135 | (Intercept) | 2.64 | 0.69 | 3.84 | 134.91 | <0.001 | 0.00 | 0.33 |
| log(CPV) ~ log(vDNE) (pooled) | 135 | x | 0.04 | 0.13 | 0.31 | 134.85 | 0.75 | 0.00 | 0.33 |
| log(CPV) ~ log(vDNE)*Tooth | 135 | (Intercept) | 3.93 | 0.66 | 5.97 | 112.50 | <0.001 | 0.28 | 0.73 |
| log(CPV) ~ log(vDNE)*Tooth | 135 | x | -0.26 | 0.13 | -2.01 | 111.94 | 0.05 | 0.28 | 0.73 |
| log(CPV) ~ log(vDNE)*Tooth | 135 | ToothM2 | 0.05 | 0.83 | 0.06 | 95.68 | 0.96 | 0.28 | 0.73 |
| log(CPV) ~ log(vDNE)*Tooth | 135 | ToothM3 | 0.02 | 1.64 | 0.01 | 103.80 | 0.99 | 0.28 | 0.73 |
| log(CPV) ~ log(vDNE)*Tooth | 135 | x:ToothM2 | 0.07 | 0.16 | 0.43 | 95.73 | 0.67 | 0.28 | 0.73 |
| log(CPV) ~ log(vDNE)*Tooth | 135 | x:ToothM3 | 0.06 | 0.31 | 0.20 | 103.85 | 0.84 | 0.28 | 0.73 |
| log(CPV) ~ log(vDNE)*Tooth simple slopes | 135 | simple slope: M1 | -0.26 | 0.13 | -1.94 | 117.95 | 0.05 | NA | NA |
| log(CPV) ~ log(vDNE)*Tooth simple slopes | 135 | simple slope: M2 | -0.19 | 0.14 | -1.32 | 125.53 | 0.19 | NA | NA |
| log(CPV) ~ log(vDNE)*Tooth simple slopes | 135 | simple slope: M3 | -0.19 | 0.29 | -0.66 | 109.14 | 0.51 | NA | NA |
| log(CPV) ~ log(DER) (pooled) | 129 | (Intercept) | 2.66 | 0.06 | 43.87 | 120.63 | <0.001 | 0.10 | 0.50 |
| log(CPV) ~ log(DER) (pooled) | 129 | x | -0.07 | 0.02 | -4.23 | 112.53 | <0.001 | 0.10 | 0.50 |
| log(CPV) ~ log(DER)*Tooth | 129 | (Intercept) | 2.45 | 0.08 | 30.51 | 128.88 | <0.001 | 0.27 | 0.80 |
| log(CPV) ~ log(DER)*Tooth | 129 | x | -0.10 | 0.04 | -2.51 | 119.15 | 0.01 | 0.27 | 0.80 |
| log(CPV) ~ log(DER)*Tooth | 129 | ToothM2 | 0.58 | 0.10 | 6.17 | 90.76 | <0.001 | 0.27 | 0.80 |
| log(CPV) ~ log(DER)*Tooth | 129 | ToothM3 | 0.59 | 0.10 | 5.96 | 90.69 | <0.001 | 0.27 | 0.80 |
| log(CPV) ~ log(DER)*Tooth | 129 | x:ToothM2 | 0.11 | 0.04 | 2.80 | 86.15 | 0.01 | 0.27 | 0.80 |
| log(CPV) ~ log(DER)*Tooth | 129 | x:ToothM3 | 0.13 | 0.04 | 3.41 | 93.12 | <0.01 | 0.27 | 0.80 |
| log(CPV) ~ log(DER)*Tooth simple slopes | 129 | simple slope: M1 | -0.10 | 0.04 | -2.42 | 125.88 | 0.02 | NA | NA |
| log(CPV) ~ log(DER)*Tooth simple slopes | 129 | simple slope: M2 | 0.01 | 0.04 | 0.37 | 130.55 | 0.71 | NA | NA |
| log(CPV) ~ log(DER)*Tooth simple slopes | 129 | simple slope: M3 | 0.03 | 0.02 | 1.17 | 126.88 | 0.24 | NA | NA |
| log(CPV) ~ log(OPCR) (pooled) | 135 | (Intercept) | 2.94 | 0.37 | 7.93 | 129.02 | <0.001 | 0.00 | 0.32 |
| log(CPV) ~ log(OPCR) (pooled) | 135 | x | -0.02 | 0.10 | -0.25 | 128.06 | 0.81 | 0.00 | 0.32 |
| log(CPV) ~ log(OPCR)*Tooth | 135 | (Intercept) | 4.16 | 0.36 | 11.43 | 103.56 | <0.001 | 0.31 | 0.78 |
| log(CPV) ~ log(OPCR)*Tooth | 135 | x | -0.42 | 0.10 | -4.28 | 101.81 | <0.001 | 0.31 | 0.78 |
| log(CPV) ~ log(OPCR)*Tooth | 135 | ToothM2 | -0.57 | 0.50 | -1.15 | 99.98 | 0.25 | 0.31 | 0.78 |
| log(CPV) ~ log(OPCR)*Tooth | 135 | ToothM3 | -1.03 | 0.73 | -1.41 | 103.41 | 0.16 | 0.31 | 0.78 |
| log(CPV) ~ log(OPCR)*Tooth | 135 | x:ToothM2 | 0.26 | 0.13 | 1.99 | 100.03 | 0.05 | 0.31 | 0.78 |
| log(CPV) ~ log(OPCR)*Tooth | 135 | x:ToothM3 | 0.37 | 0.19 | 1.95 | 103.36 | 0.05 | 0.31 | 0.78 |
| log(CPV) ~ log(OPCR)*Tooth simple slopes | 135 | simple slope: M1 | -0.42 | 0.10 | -4.15 | 107.84 | <0.001 | NA | NA |
| log(CPV) ~ log(OPCR)*Tooth simple slopes | 135 | simple slope: M2 | -0.16 | 0.11 | -1.49 | 116.43 | 0.14 | NA | NA |
| log(CPV) ~ log(OPCR)*Tooth simple slopes | 135 | simple slope: M3 | -0.05 | 0.16 | -0.31 | 109.50 | 0.76 | NA | NA |
| log(CPV) ~ log(RFI) (pooled) | 135 | (Intercept) | 3.27 | 0.21 | 15.35 | 128.22 | <0.001 | 0.03 | 0.38 |
| log(CPV) ~ log(RFI) (pooled) | 135 | x | 0.37 | 0.18 | 2.00 | 124.84 | 0.05 | 0.03 | 0.38 |
| log(CPV) ~ log(RFI)*Tooth | 135 | (Intercept) | 3.02 | 0.33 | 9.25 | 113.96 | <0.001 | 0.29 | 0.76 |
| log(CPV) ~ log(RFI)*Tooth | 135 | x | 0.34 | 0.27 | 1.27 | 111.96 | 0.21 | 0.29 | 0.76 |
| log(CPV) ~ log(RFI)*Tooth | 135 | ToothM2 | -0.36 | 0.39 | -0.93 | 94.88 | 0.35 | 0.29 | 0.76 |
| log(CPV) ~ log(RFI)*Tooth | 135 | ToothM3 | -0.95 | 0.39 | -2.41 | 94.65 | 0.02 | 0.29 | 0.76 |
| log(CPV) ~ log(RFI)*Tooth | 135 | x:ToothM2 | -0.63 | 0.32 | -1.94 | 95.14 | 0.06 | 0.29 | 0.76 |
| log(CPV) ~ log(RFI)*Tooth | 135 | x:ToothM3 | -1.17 | 0.35 | -3.32 | 94.95 | <0.01 | 0.29 | 0.76 |
| log(CPV) ~ log(RFI)*Tooth simple slopes | 135 | simple slope: M1 | 0.34 | 0.27 | 1.23 | 118.17 | 0.22 | NA | NA |
| log(CPV) ~ log(RFI)*Tooth simple slopes | 135 | simple slope: M2 | -0.29 | 0.27 | -1.07 | 122.30 | 0.29 | NA | NA |
| log(CPV) ~ log(RFI)*Tooth simple slopes | 135 | simple slope: M3 | -0.84 | 0.31 | -2.67 | 118.52 | 0.01 | NA | NA |
| log(CPV) ~ log(Summed Krueger-Scott scores) (pooled) | 133 | (Intercept) | 3.52 | 0.22 | 16.34 | 125.10 | <0.001 | 0.06 | 0.47 |
| log(CPV) ~ log(Summed Krueger-Scott scores) (pooled) | 133 | x | -0.23 | 0.07 | -3.13 | 121.47 | <0.01 | 0.06 | 0.47 |
| log(CPV) ~ log(Summed Krueger-Scott scores)*Tooth | 133 | (Intercept) | 3.77 | 0.43 | 8.75 | 122.45 | <0.001 | 0.27 | 0.80 |
| log(CPV) ~ log(Summed Krueger-Scott scores)*Tooth | 133 | x | -0.36 | 0.14 | -2.66 | 121.28 | 0.01 | 0.27 | 0.80 |
| log(CPV) ~ log(Summed Krueger-Scott scores)*Tooth | 133 | ToothM2 | -0.79 | 0.48 | -1.63 | 91.18 | 0.11 | 0.27 | 0.80 |
| log(CPV) ~ log(Summed Krueger-Scott scores)*Tooth | 133 | ToothM3 | -1.24 | 0.40 | -3.12 | 96.13 | <0.01 | 0.27 | 0.80 |
| log(CPV) ~ log(Summed Krueger-Scott scores)*Tooth | 133 | x:ToothM2 | 0.37 | 0.16 | 2.35 | 91.93 | 0.02 | 0.27 | 0.80 |
| log(CPV) ~ log(Summed Krueger-Scott scores)*Tooth | 133 | x:ToothM3 | 0.51 | 0.13 | 3.99 | 92.76 | <0.001 | 0.27 | 0.80 |
| log(CPV) ~ log(Summed Krueger-Scott scores)*Tooth simple slopes | 133 | simple slope: M1 | -0.36 | 0.14 | -2.57 | 127.88 | 0.01 | NA | NA |
| log(CPV) ~ log(Summed Krueger-Scott scores)*Tooth simple slopes | 133 | simple slope: M2 | 0.01 | 0.16 | 0.05 | 133.73 | 0.96 | NA | NA |
| log(CPV) ~ log(Summed Krueger-Scott scores)*Tooth simple slopes | 133 | simple slope: M3 | 0.15 | 0.09 | 1.64 | 132.25 | 0.10 | NA | NA |

**SOM Table 8.** Likelihood-ratio test results comparing tooth-only models with models including an interaction between each predictor and tooth.

| **Predictor** | **n** | **Chisq** | **p.value** |
| --- | --- | --- | --- |
| log(CPV)~log(Wear age) | 135 | 1.91 | 0.59 |
| log(CPV)~log(Convex DNE) | 135 | 5.05 | 0.168 |
| log(CPV)~log(OPCR) | 135 | 17.4 | 0.000575 |
| log(CPV)~log(RFI) | 135 | 11.4 | 0.00963 |
| log(CPV)~log(Summed Krueger-Scott scores) | 133 | 14.9 | 0.0019 |
| log(CPV)~log(DER) | 129 | 11.5 | 0.00949 |

**SOM Table 9.** Semi-partial marginal R^2^ values and confidence intervals for the contributions of each variable to CPV in a multivariate model.

| **Predictor(s)** | **R^2^** | **CI_lower** | **CI_upper** |
| --- | --- | --- | --- |
| Model | 0.172 | 0.1043 | 0.3543 |
| Log(wear age) | 0.055 | 0 | 0.2417 |
| Log(DER) | 0.033 | 0 | 0.2195 |
| Log (Summed Krueger-Scott scores) | 0 | 0 | 0.1896 |
| Log(OPCR) | 0.012 | 0 | 0.2004 |
| Log(RFI) | 0.0044 | 0 | 0.194 |
| Log(vDNE) | 0 | 0 | 0.1785 |
